# Redescription and Molecular Characterization of the External Attaching Fish Parasitic Cymothoid, *Nerocila phaiopleura* Bleeker, 1857 (Crustacea: Isopoda) off the Southwest Coast of India

**DOI:** 10.1007/s11686-024-00870-7

**Published:** 2024-08-27

**Authors:** Amurtha Shyla Suresh, Balamurali Raghavan Pillai Sreekumaran Nair, Binumon Thankachan Mangalathettu, Panakkool Thamban Aneesh

**Affiliations:** 1 Post Graduate Department of Zoology and Research Centre, Mahatma Gandhi College, Pattom PO, Thiruvananthapuram, 695004 Kerala India; 2 Department of Zoology, University of Kerala, Karyavattom, Thiruvananthapuram, 695581 Kerala India; 3 Fisheries Laboratory, Blue Innovation Division, Seto Inland Sea Carbon-Neutral Research Center, Hiroshima University, 5-8-1 Minato-Machi, Takehara, 725-0024 Hiroshima Japan; 4 Travancore Nature History Society (TNHS), MBRRA, Mathrubhumi Road, Vanchiyoor, Trivandrum, Kerala 695035 India

**Keywords:** Fish parasites, Isopods, Redescription, Molecular characterization, Cymothoidae

## Abstract

**Purpose:**

The identification of the external attaching fish parasitic cymothoid, *Nerocila phaiopleura* Bleeker 1857, is still based on the brief description of Australian specimens provided by Bruce (1987). The present study aimed to provide a redescription and molecular characterisation of Indian specimens of *N. phaiopleura*.

**Materials and Methods:**

Morphological identification was carried out based on microscopic examinations and taxonomic drawings. mitochondrial DNA cox1 was selected as the target gene for sequencing and molecular identification. Nucleotide genetic divergence (p-distance) and base-pair differences among the different species were determined using MEGA11.

**Results:**

*Nerocila phaiopleura* can be well separated from its congeners by the following combination of characteristics: Body about 2.4 times as long as wide, cephalon broadly rounded anteriorly; coxae posteriorly directed, acute and extending beyond their corresponding pereonite; pereonite 7 posterior angle produced, extending to the pleonite 1; pleonites 1 and 2 ventrolateral process posteriorly directed; uropod exopod straight and elongate, 1.7–2.0 times longer than endopod; uropod endopod lateral margin not serrate, no notch on medial margin; pereopods with short ischium; pleotelson triangular. The p-distance among *N. phaiopleura* and other available *Nerocila* spp. ranged from 21 to 19%.

**Conclusion:**

This study represents the first detailed taxonomic redescription of Indian specimens of *N. phaiopleura*. Key taxonomic features of the life stages and molecular data are provided here to identify the species properly. Interspecific genetic divergence between *N. phaiopleura* and other *Nerocila* spp. is assessed for the first time. Studies in cymothoid life histories, genetics, and morphology are necessary to understand one of the least understood parasite families.

## Introduction

The external attaching fish parasitic cymothoid genus *Nerocila* Leach, 1818 comprises 57 valid species [1] and is associated with many commercially important fish species worldwide [2]. Several species of *Nerocila* are morphologically highly variable, and their identification is often difficult [3, 4]. Notwithstanding the few recent reports, many species of *Nerocila* still require revision, including *Nerocila phaiopleura* Bleeker, 1857. Twelve valid species of *Nerocila* (*N. loveni* Bovallius, 1887, *N. exocoeti* Pillai, 1954, *N. trichiura* Miers, 1878*, N. serra* Schioedte and Meinert, 1881*, N. depressa* H. Milne Edwards, 1840, *N. poruvae* Rameshkumar, Ravichandran and Trilles*,* 2011, *N. recurvispina* Schioedte and Meinert, 1881, *N. longispina* Miers, 1880*, N. arres* Bowman and Tareen, 1983*, N. phaiopleura* Bleeker, 1857*, N. sigani* Bowman and Tareen, 1983 and *N. sundaica* Bleeker, 1857) have been known from Indian waters [5–7].

*Nerocila phaiopleura* is a widely distributed and variable species found as an external parasite attaching to the skin of nearly 50 species of fishes belonging to 18 families, including Clupidae, Engraulidae, Carangidae, Scombridae, Dussumieriidae, Chirocentridae, Pristigasteridae, Mugilidae, Sphyraenidae, Leiognathidae, Plotosidae, Polynemidae, Ariidae and Istiophoridae from the Indian and Pacific oceans [8–12]. The species has been recorded from about 20 different fish species from India [10, 13].

Bleeker [14] provided the original description of *N. phaiopleura*. Pillai [8] gave brief descriptions of the species with illustrations of only a dorsal view of the species. Subsequently, Bruce [3] provided a short description of Australian specimens of *N. phaiopleura,* and after this, no detailed taxonomic information on the species was available. The previous reports of this species were brief and required further clarification on the identity. There is no previous taxonomic description for the moulting stage of *N. phaiopleura*.

The taxonomic status of many parasitic isopod species is still confusing, emphasizing the significance of detailed descriptions with accurate illustrations. The identification of *N. phaiopleura* is still based on the descriptions mentioned above. A detailed description based on Indian specimens is highly needed; since the Indian specimen is not adequately described yet. The present study provides molecular characterization and a detailed morphological description of the female and moulting stages of the parasitic isopod, *N. phaiopleura*, collected from the two host fishes *Dussumieria acuta* Valenciennes, 1847 and *Rastrelliger kanagurta* Cuvier, 1816 from the Kerala coast, India.

## Materials and Methods

The specimens of *Nerocila phaiopleura* Bleeker 1857 were collected from the host fish (*Dussumieria acuta* Valenciennes, 1847 and *Rastrelliger kanagurta* Cuvier, 1816) in Neendakara fishing harbour (Lat. 8°56′19" N, Long. 76°32′25" E), Southwest coast of India. The mode of attachment of isopods to the host skin and the gross changes they made were observed. The parasites isolated were cleaned (removing mucus and other debris adhering to their bodies) and preserved in 70% alcohol/10% Neutral Buffered formalin for taxonomic studies. A few samples were preserved in 100% ethanol for genetic analysis.

### Morphological Identification

For morphological identification, the parasite's general morphological features were studied using a hand lens and under a Stereo Dissection Microscope (SDM) (Carl Zeiss Microscopy; GmbH Stemi 508). Later, the parasites were dissected in 50% lactic acid; taxonomically important body parts such as pereopods, pleopods, uropods and mouthparts were carefully dissected according to the techniques described by Aneesh et al. [6, 15–17]. The parasites' total length and maximum width was measured to the nearest millimetre using a Vernier calliper. The species identification followed Bruce [3, 4]. Sources for the fish taxonomy and host nomenclature were based on Fish Base [18] and Catalogue of Fishes [19].

The mode of attachment of isopods to the host skin and the gross changes made by the parasite were photographed using a Canon EOS 800D with a 35 mm macro lens. Microphotographs of the parasite and its body parts were taken using SDM. The drawings of the body parts of the parasite were made with a drawing tube attached to the Transmission Light Microscope (TLM) (Optika Microscope; Optikam B5 Digital Camera). Taxonomic drawings were made using CorelDraw software (CorelDRAW X7). The voucher specimens are deposited in the Western Ghat Field Research Centre of Zoological Survey of India, Kozhikode (ZSI/WGRC).

### Molecular Analysis

Genomic DNA was isolated from the tissues following the protocol for animal tissue extraction using NucleoSpin® Tissue Kit (Macherey–Nagel, Düren, Germany) following the manufacturer's instructions. A targeted part of the mitochondrial cytochrome *c* oxidase subunit I (COI) gene (approximately 680 bp) of these specimens was subjected to PCR amplification with the aid of a PCR thermal cycler (GeneAmp PCR System 9700, Applied Biosystems) and universal invertebrate primers LCO1490 (5′-GGTCAACAAATCATAAAGATATTGG-3′) and HC02198 (5′-TAAACTTCAGGGTGACCAAAAAATCA-3′).

PCR reactions were performed with volumes of 10.5 μl, using 5 μl 2 × Phire Master Mix, 0.25 μl of each primer, 4 μl of PCR–grade nuclease-free water and 1 μl of DNA. Conditions for the PCR were as follows: initial denaturation at 98 °C for 30 s, followed by ten cycles of 98°C for 5 s, annealing at 45 °C for 10 s with an end extension at 72 °C for 15 s, followed by 30 cycles of 98°C for 5 s, annealing at 50 °C for 10 s with an end extension at 72 °C for 15 s, and ending with a final extension of 72 °C for 60 s.

The sequencing reaction was done in a PCR thermal cycler (GeneAmp PCR System 9700, Applied Biosystems) using the BigDye Terminator v3.1 Cycle sequencing Kit (Applied Biosystems, USA) following the manufacturer's protocol. The cleaned-up air-dried product was sequenced in ABI 3500 DNA Analyzer (Applied Biosystems). The sequence quality was checked using Sequence Scanner Software v1 (Applied Biosystems). Sequence alignment and required editing of the obtained sequences were carried out using Geneious Pro v5.1 [20].

Comparative sequences of *Nerocila* species from GenBank were downloaded and aligned to one sequence. These sequences included: OP890359 (*N. phaiopleura*), KY933655 (*N. loveni*), ON661340 (*N. exocoeti*), OK001962 (*N. longispina*), LC160331 *(N. japonica*) and MZ644982 (*N. orbignyi*). Nucleotide genetic divergence (p-distance) and base-pair differences among the different species were determined using MEGA11 [21]. Maximum Composite Likelihood method is used for p-distance calculation.

## Results

### Taxonomy

Suborder Cymothoida Wägele, 1989

Superfamily Cymothooidea Leach, 1814

Family Cymothoidae Leach, 1814

Genus *Nerocila* Leach, 1818

*Nerocila phaiopleura* Bleeker, 1857

(Figs. 1, 2, 3, 4, 5, 6, 7, 8, 9, 10).

**Fig. 1:**
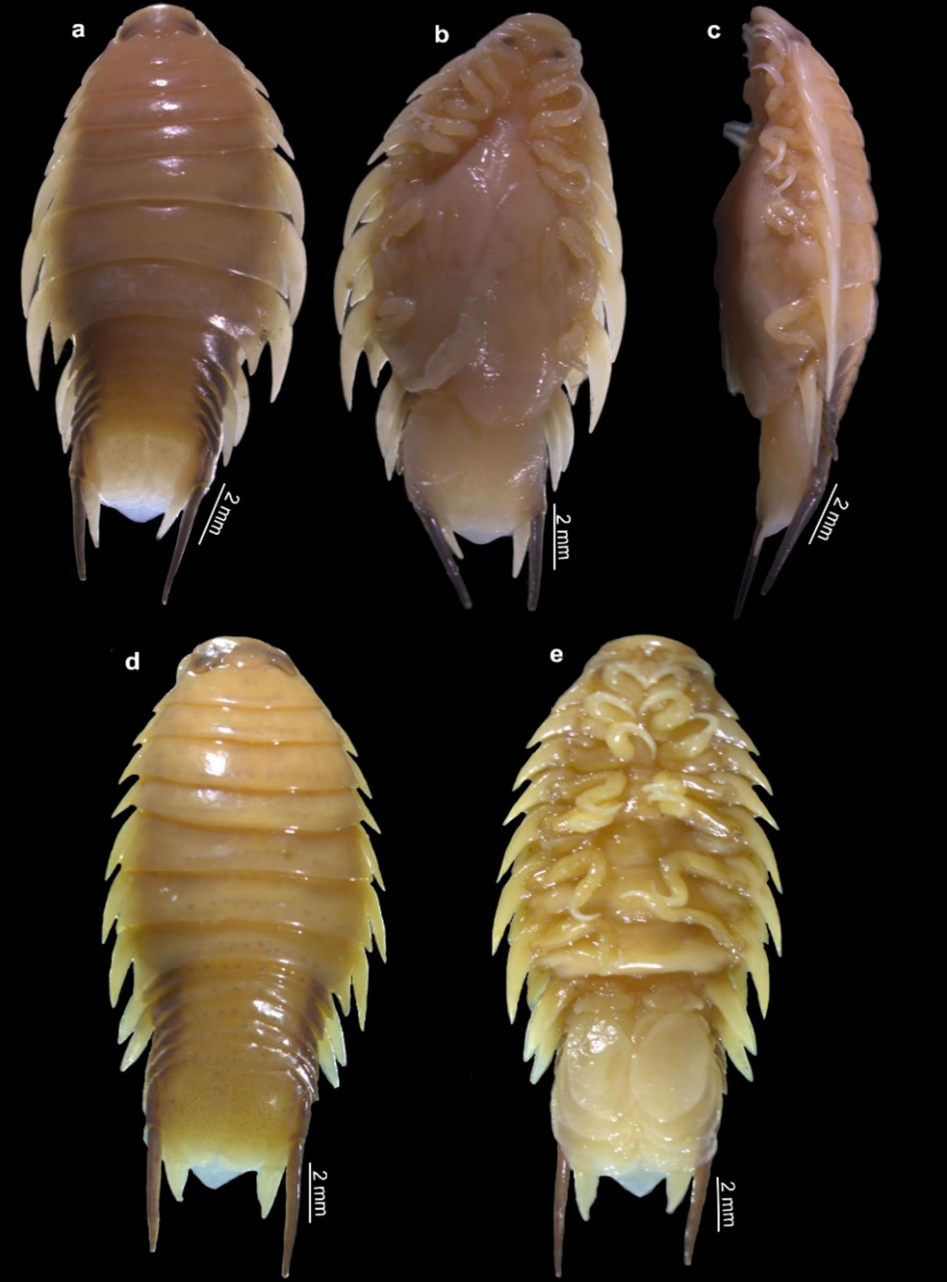
*Nerocila phaiopleura* Bleeker 1857 from *Rastrelliger kanagurta* Cuvier (**a**-**c**) Ovigerous female dorsal, ventral, and lateral view, respectively (**d**, **e**) non-ovigerous female dorsal and ventral view, respectively

**Fig. 2:**
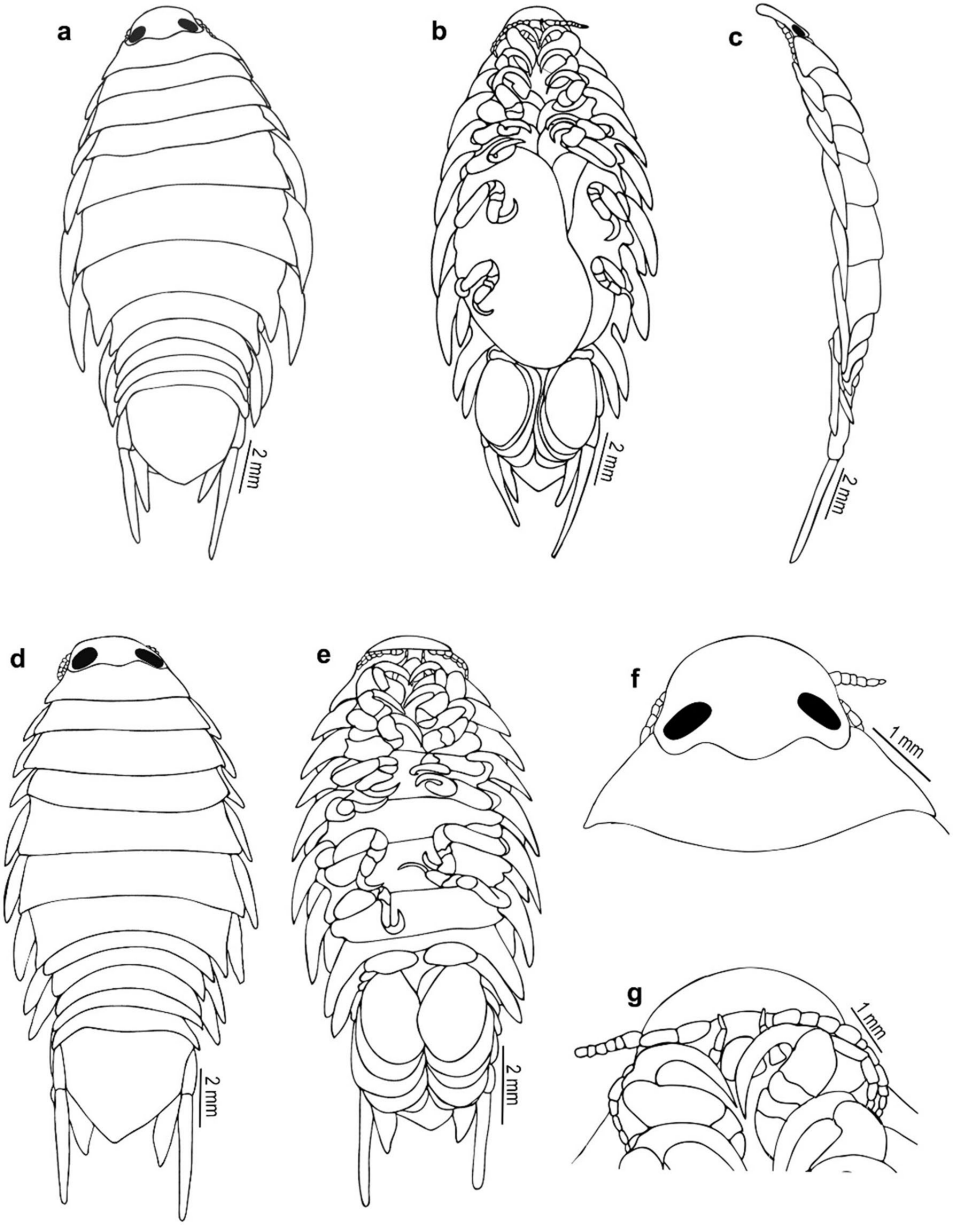
*Nerocila phaiopleura* Bleeker 1857 from *Rastrelliger kanagurta* Cuvier (**a**–**c**) Ovigerous female dorsal, ventral, and lateral view, respectively (**d** and **e**) Non-ovigerous female, dorsal and ventral view respectively (**f** and **g**) Ovigerous female cephalon, dorsal and ventral view, respectively

**Fig. 3:**
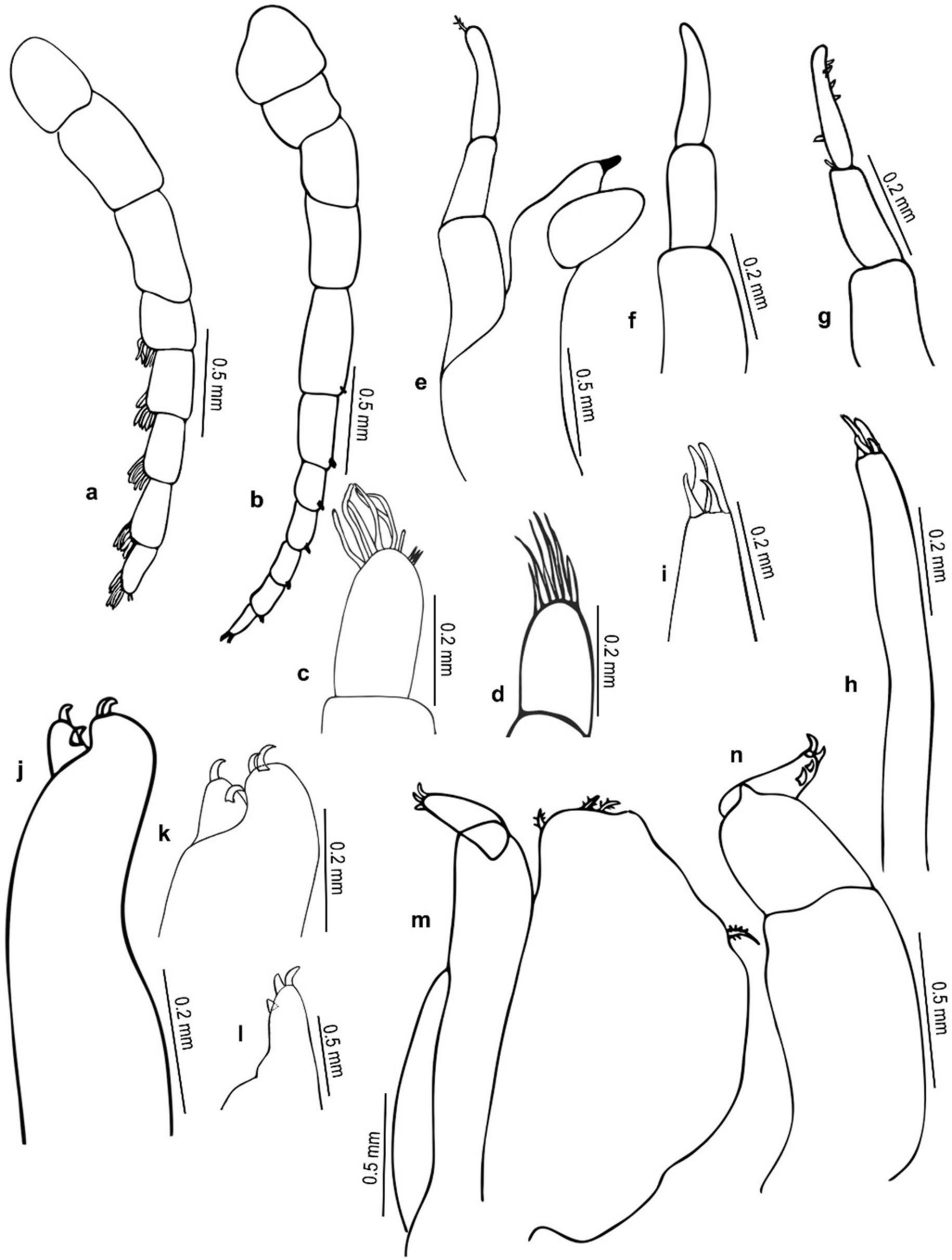
*Nerocila phaiopleura* Bleeker 1857 from *Rastrelliger kanagurta* Cuvier, female (**a**–**m**) Ovigerous female mouth parts (**a**) Antennula (**b**) Antenna (**c**) Antenna apex (**d**) Antennule apex (**e**) Mandible (**f** and **g**) Mandibular palp variations (**h**) Maxillule (**i**) Maxillule apex (**j**) Maxilla (**k**) Maxilla apex (**l**) Maxilliped apex (**m**) Maxilliped (**n**) Non-ovigerous female, maxilliped

**Fig. 4:**
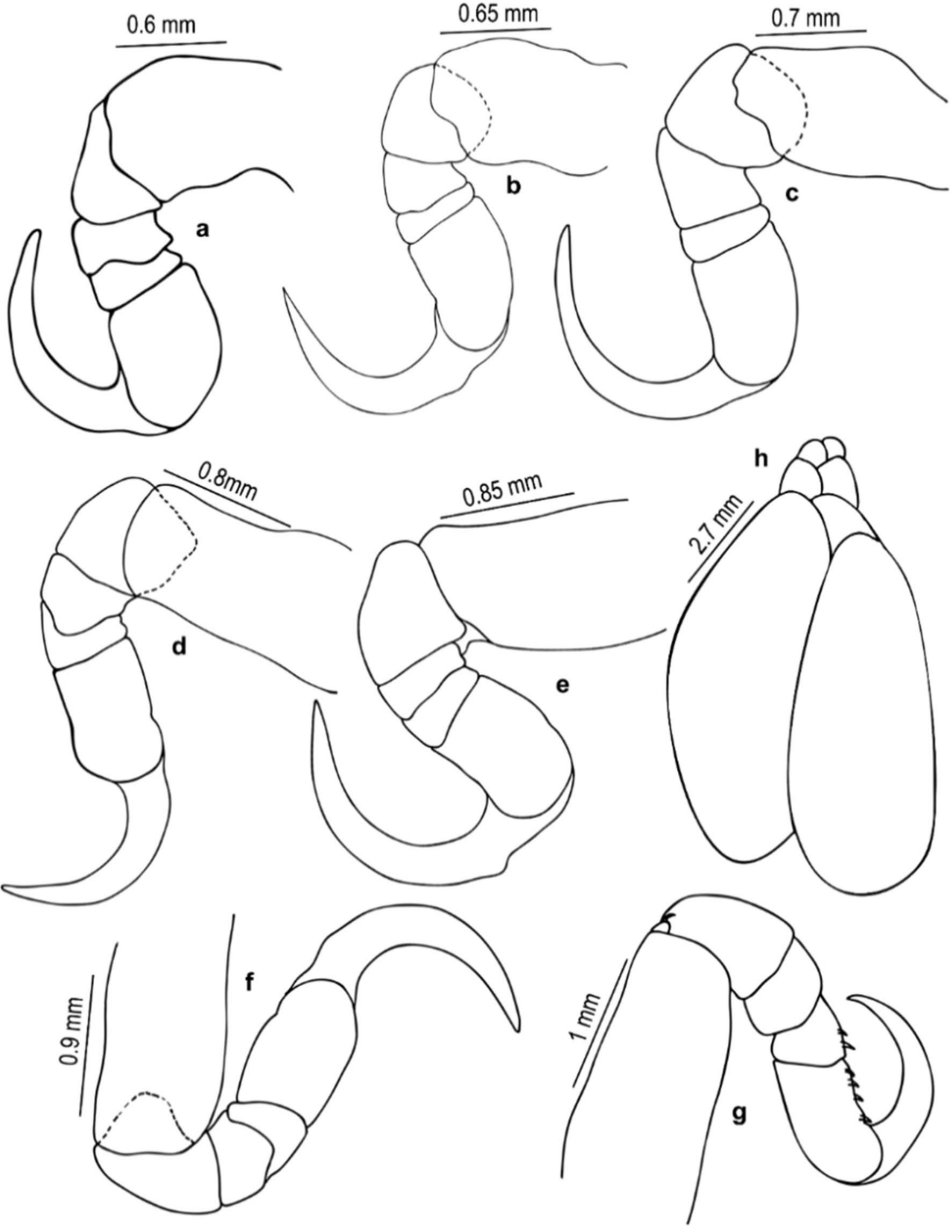
*Nerocila phaiopleura* Bleeker 1857 from *Rastrelliger kanagurta* Cuvier, ovigerous female (**a**–**g**) Pereopod 1–7, respectively (**h**) Oostegite

**Fig. 5:**
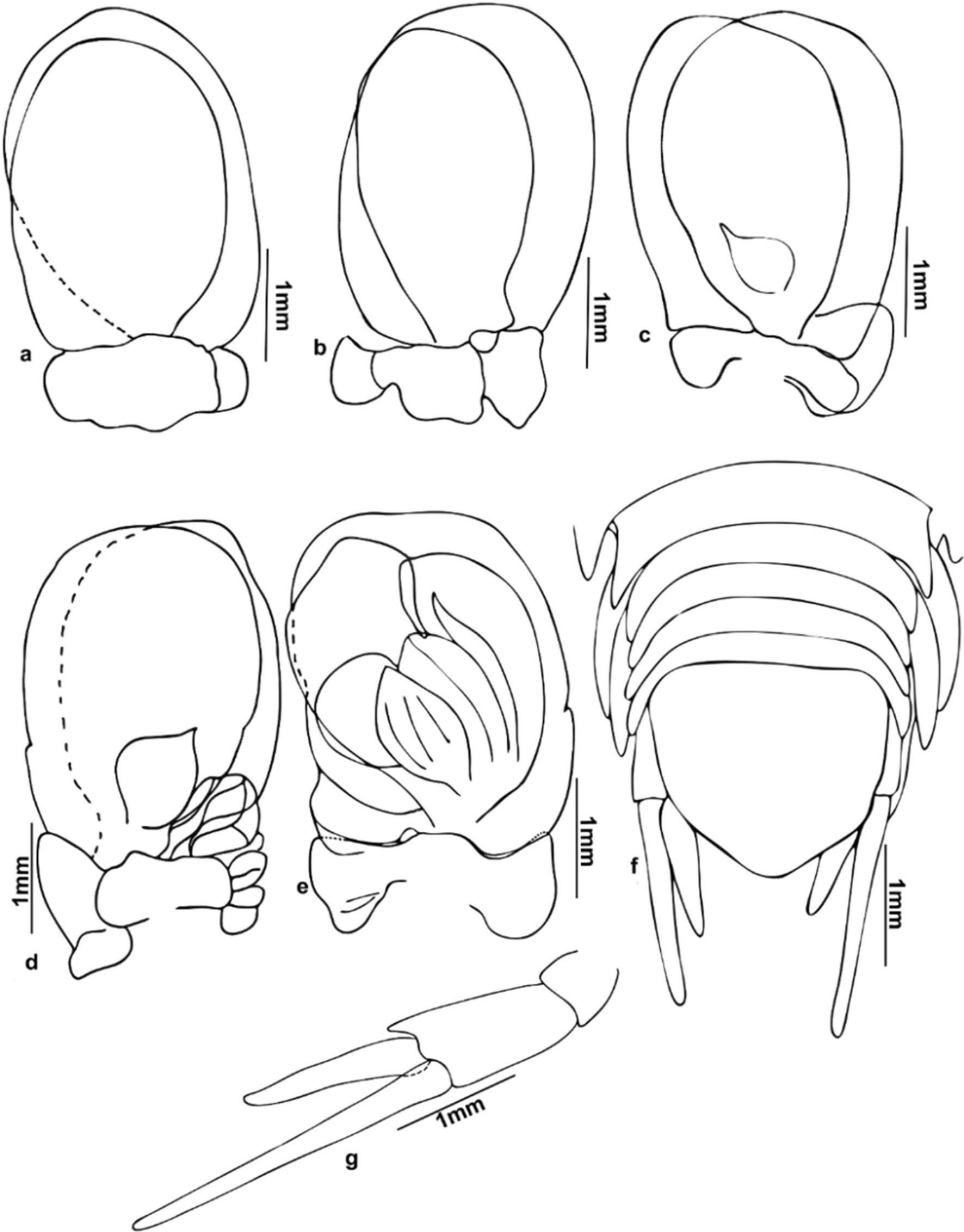
*Nerocila phaiopleura* Bleeker 1857 from *Rastrelliger kanagurta* Cuvier, ovigerous female (**a**–**e**) Pleopod 1–5, respectively

**Fig. 6:**
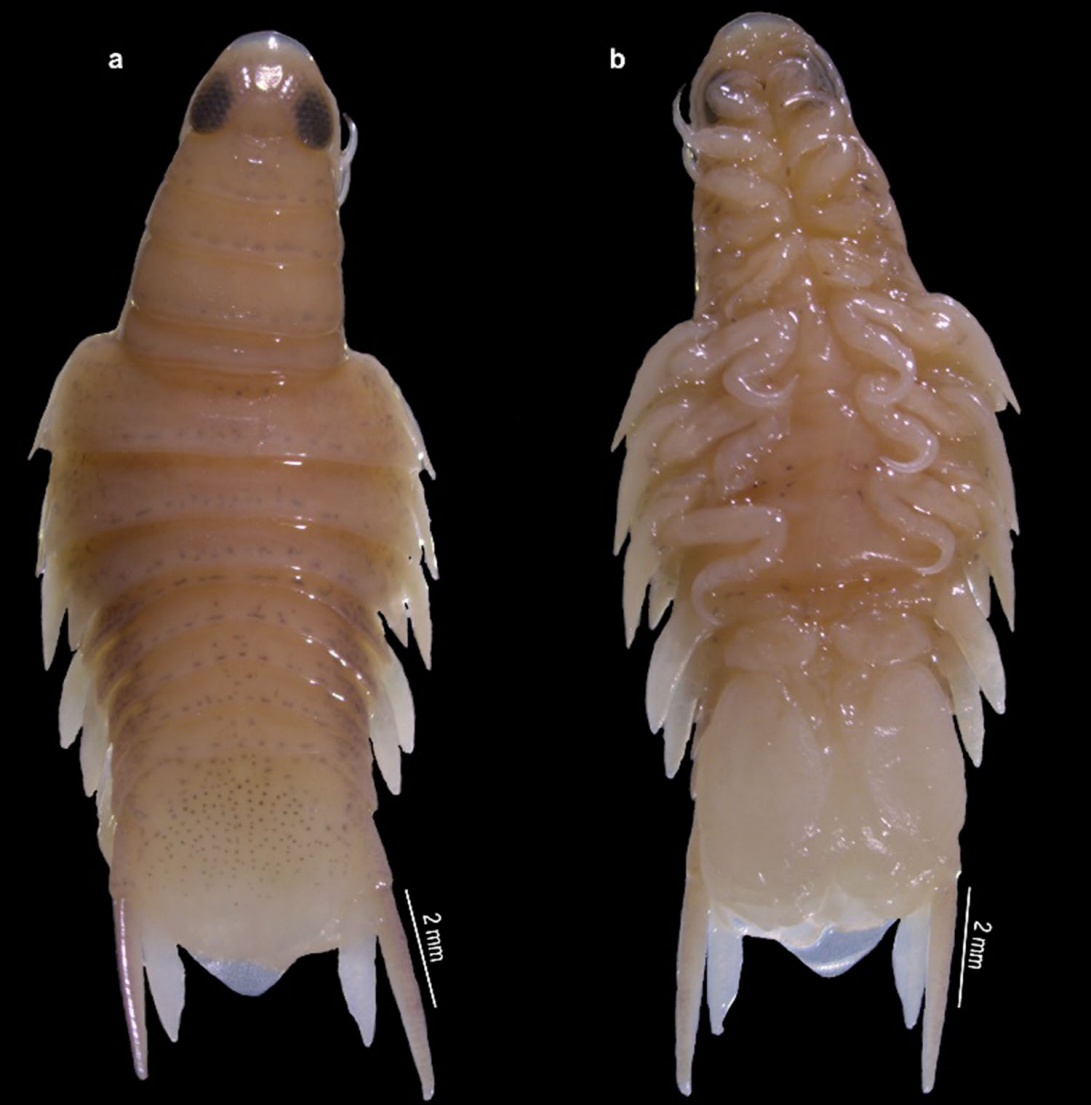
*Nerocila phaiopleura* Bleeker 1857 from *Rastrelliger kanagurta* Cuvier, partially moulted male, (**a** and **b**) Dorsal and ventral view, respectively

**Fig. 7:**
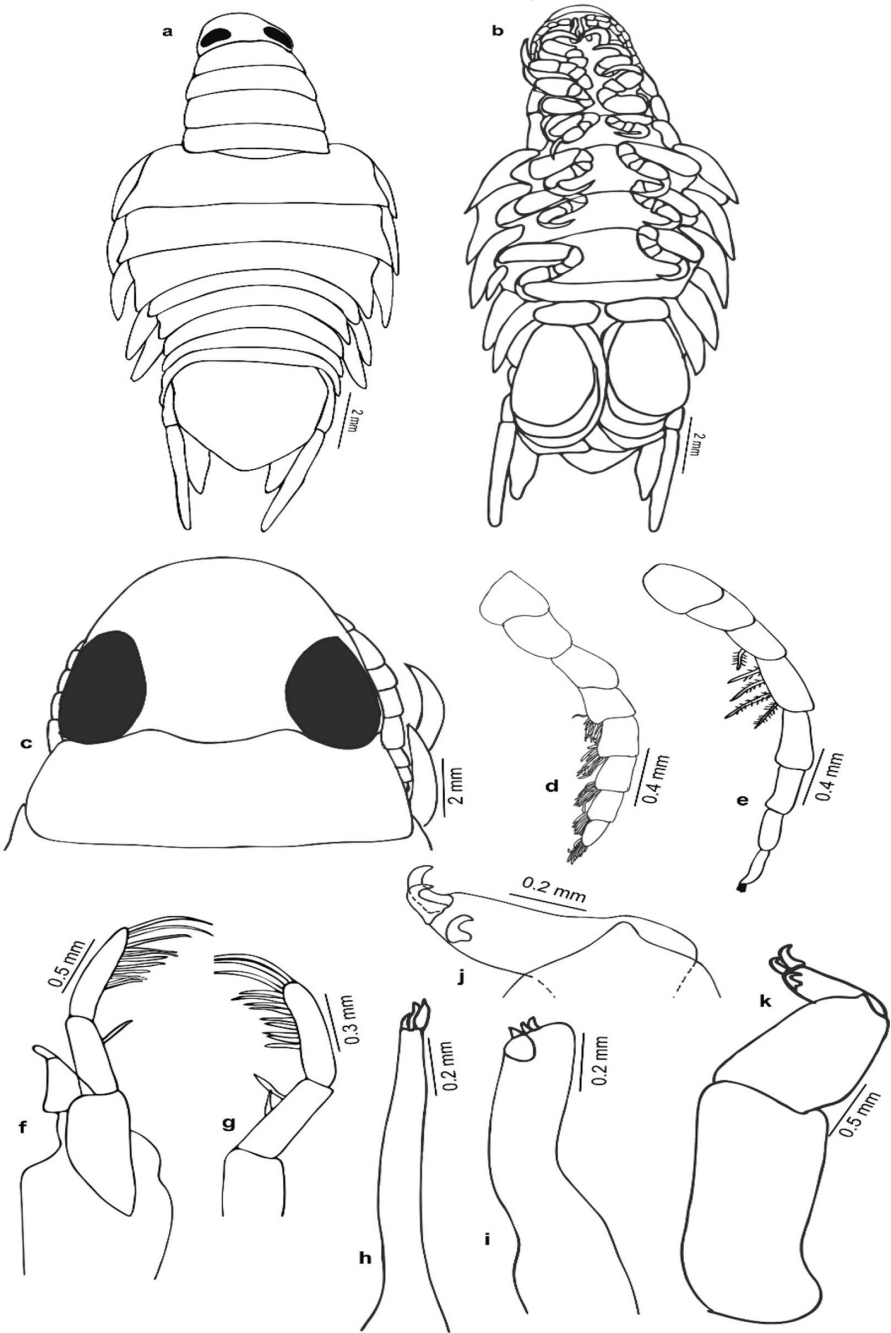
*Nerocila phaiopleura* Bleeker 1857 from *Rastrelliger kanagurta* Cuvier, male moulting stage, (**a** and **b**) Dorsal and ventral view respectively (**c**) Cephalon (**d**) Antennula (**e**) Antenna (**f**) Mandible (**g**) Mandibular palp variation (**h**) Maxillule (**i**) Maxilla (**j**) Maxilliped apex (**k**) Maxilliped

**Fig. 8:**
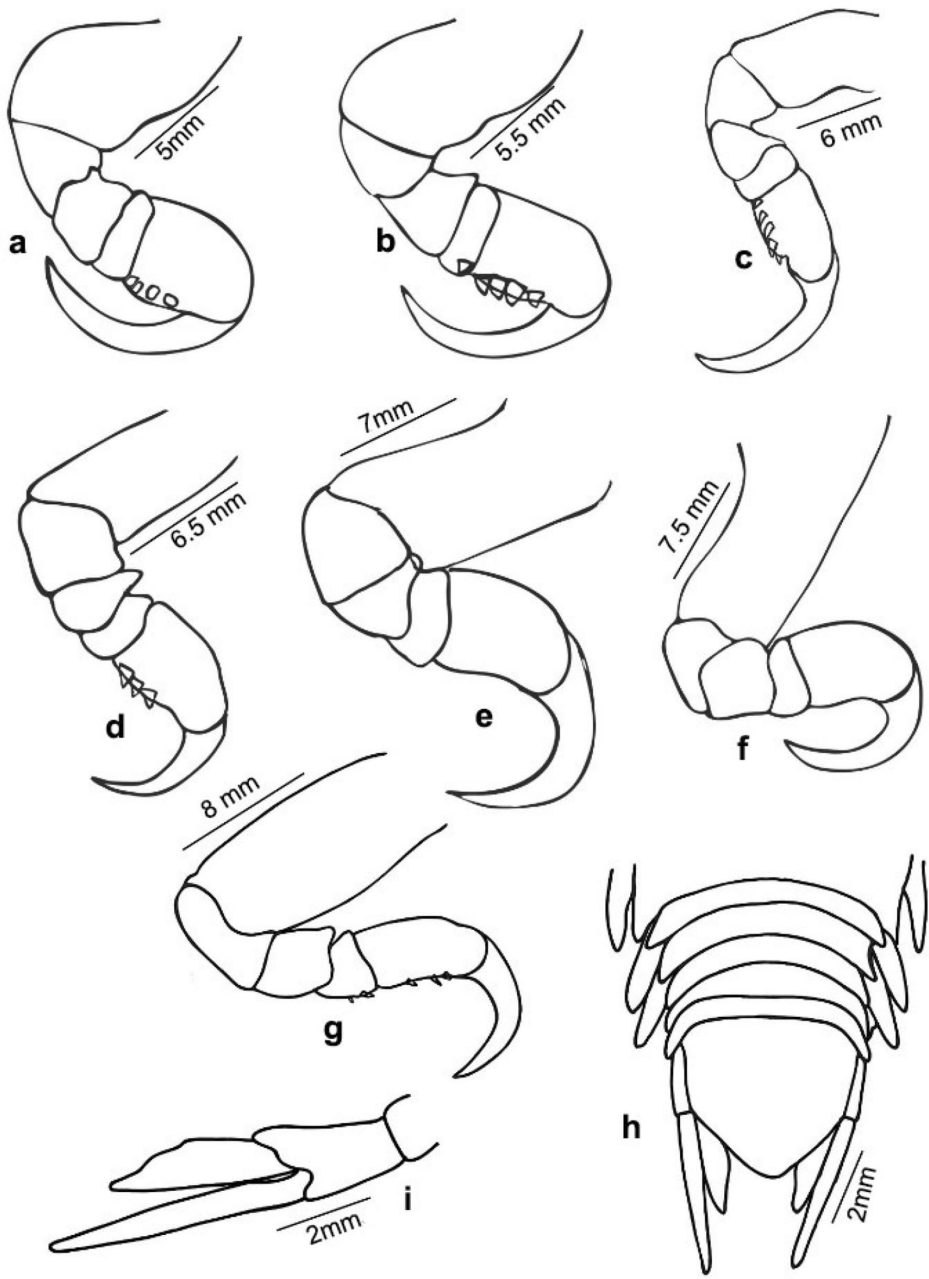
*Nerocila phaiopleura* Bleeker 1857 from *Rastrelliger kanagurta* Cuvier, male moulting stage, (**a**–**g**) Pereopods 1–7, respectively (**h**) Pleotelson (**i**) Uropod

**Fig. 9:**
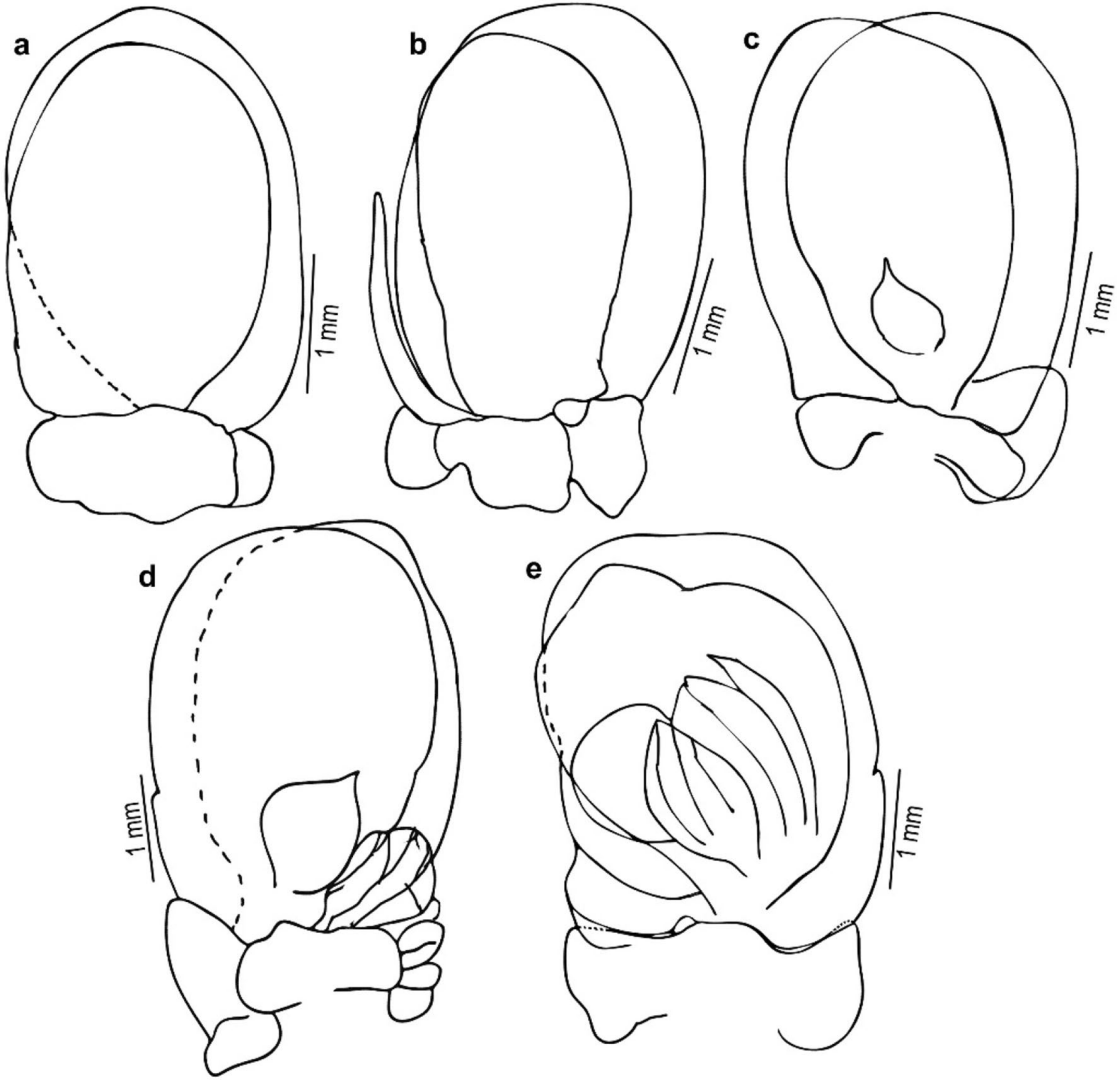
*Nerocila phaiopleura* Bleeker 1857 from *Rastrelliger kanagurta* Cuvier, male moulting stage (**a**–**e**) Pleopod 1–5 respectively

**Fig. 10:**
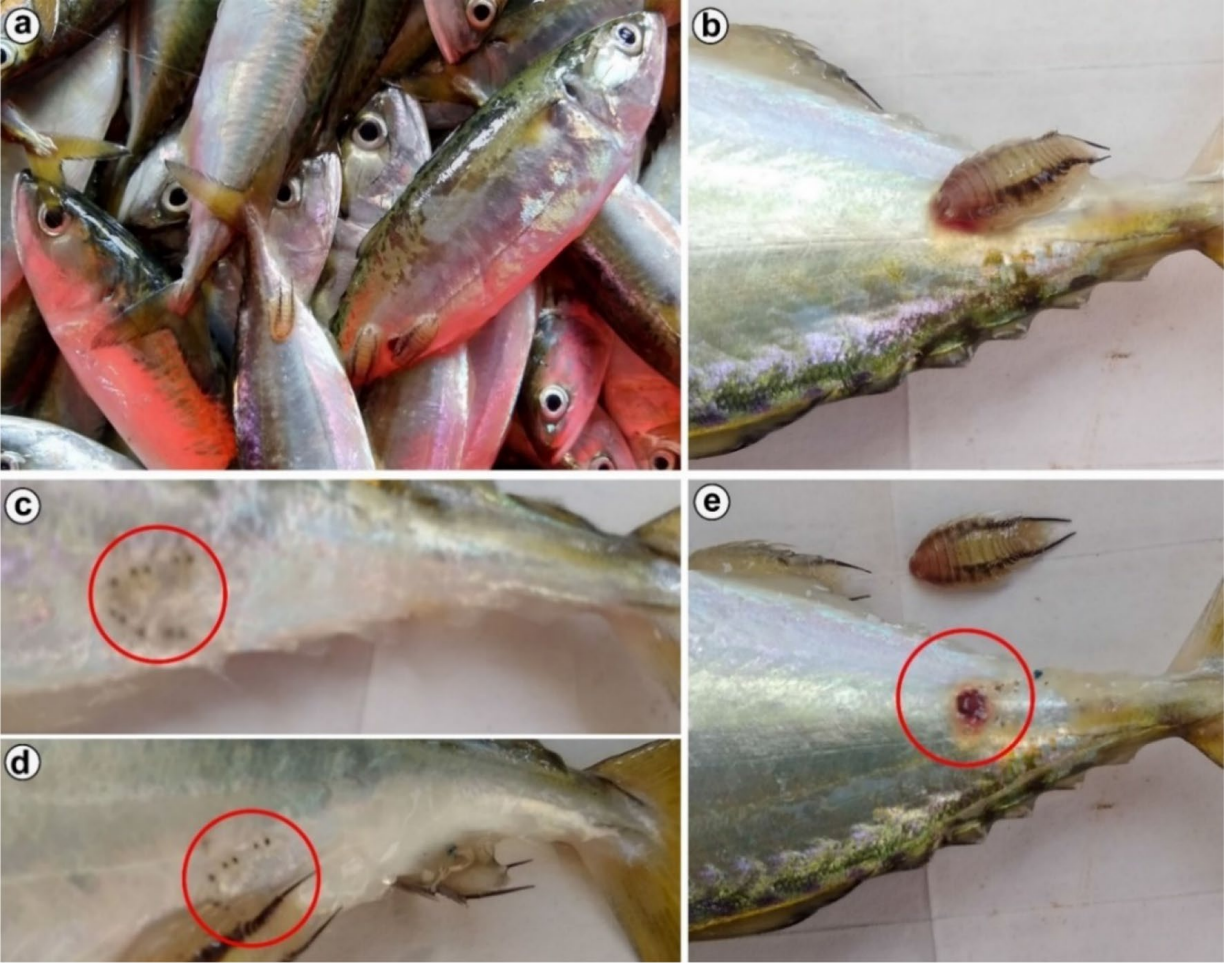
*Nerocila phaiopleura* Bleeker 1857 infestation on host, *Rastrelliger kanagurta* Cuvier (**a**) Heavy infestation of the parasite on the host (**b**) Parasite attachment and feeding (**c** and **d**) Small pin holes with the formation of epidermal plaques at the attachment site of parasite (red arrows) (**e**) Large haemorrhagic wound made by the parasite due to the attachment and feeding activities

*Nerocila phaiopleura* Bleeker, 1857: 25, pl. 1 (Fig. 3)—Monod, 1976: 857—Trilles, 1979: 253, pl. 1 (Fig. 3).

*Nerocila phaeopleura*—Miers, 1880: 467;—Schiöedte & Meinert, 1881: 13, pl. 1 (Figs. 6, 7)—Gerstaecker, 1882: 260.—Nierstrasz, 1915: 75, pl. 3 (Figs. 1, 2); 1918: 113, pl. 9 (Figs. 6, 7); 1931: 124—Barnard, 1925: 392.—Chilton, 1926: 180, Fig. 3a–b.—Monod, 1934: 12.—Serene, 1937: 69—Morton, 1974: 143, pl. 1—Kensley, 1978: 82, Fig. 33d, g

*Nerocila* (*Nerocila*) *phaeopleura*—Bruce, 1982: 316, Figs. 1, 4a–c

*Nerocila* (*Nerocila*) *phaiopleura* Bowman & Tareen, 1983: 5, Fig. 5

*Nerocila phaeopleura* [sic]—Barnard, 1936: 164, Fig. 6a–c

*Nerocila* spp. Monod, 1976: 857, Figs. 14, 15.—Meenakshisundaram, 1965: 202–204, Figs 1, 2.—Seshagiri Rao, 1974: 428— Ranjitsingh & Padmalatha, 1997: 171, Figs. 1, 2.—Radhakrishnan & Nair, 1983: 93–115, Fig. 10d

*Nerocila phaiopleura*—Bruce, 1987b: 384, Figs 18, 19.—Bruce & Harrison-Nelson, 1988: 596.—Ravichandran, Ranjith Singh & Veerappan, 2001: 622–623, Figs. 1, 2.—Kazmi, Schotte & Yousuf, 2002: 103, Fig. 84.—Trilles, Ravichandran & Rameshkumar, 2011: 452.—Trilles, Rameshkumar & Ravichandran, 2013: 1273–1286, Fig. 2f.—Bharadhirajan, Murugan, Sakthivel & Selvakumar, 2014: 268–272, Fig. 2a–d.—Rameshkumar, Ramesh, Ravichandran & Trilles, 2014c: 940–944, Fig. 1g.—Rameshkumar, Ravichandran & Ramesh, 2014: 124–128, Fig. 5.—Ravichandran, Sivasubramanian, Parasuraman, Karthick Rajan & Rameshkumar, 2016a: 1–5, Figs 1, 3.—Ravichandran, Vigneshwaran & Rameshkumar, 2019: 58, Fig. 8j–l.

*Material examined: Voucher specimens:* All materials from *Rastrelliger kanagurta* (Scombridae), off Neendakara coast, 08°30.0’N, 76°53.30’E, coll. Amrutha SS on December, 2021. 1 ovigerous female (22.5 mm L; 9.5 mm W) (Reg. No. ZSI/WGRC/I.R-INV 27087); 1 ovigerous female (22 mm L; 9.5 mm W) (Reg. No.  ZSI/WGRC/I.R-INV. 27088); 1 ovigerous female (20 mm L; 8.5 mm W) (Reg. No.  ZSI/WGRC/I.R-INV. 27089); 1 ovigerous female (20 mm L; 9 mm W) (Reg. No  ZSI/WGRC/I.R-INV. 27090); 1 ovigerous female (21 mm L; 9.5 mm W) (Reg. No.  ZSI/WGRC/I.R-INV. 27091); 1 non-ovigerous female (19 mm L; 8 mm W) (Reg. No. ZSI/WGRC/I.R-INV. 27092); 1 non-ovigerous female (20.5 mm L; 8 mm W) (Reg. No.); 1 non-ovigerous female (17 mm L; 6.5 mm W) (Reg. No. ZSI/WGRC/I.R-INV. 27093); 1 transitional stage (male moulting stage) (15.5 mm L; 5.5 mm W) (Reg. No. ZSI/WGRC/I.R-INV. 27094); 1 transitional stage (male moulting stage) (16.5 mm L; 7 mm W) (Reg. No ZSI/WGRC/I.R-INV. 27096).

***Redescription of ovigerous female**** (*Figs. 1, 2, 3, 4, 5*): Body* ovate, about 2.4 times as long as wide, widest at pereonite 5–6. *Cephalon* broadly rounded anteriorly, 1.6 times wider than long, posterior margin prominently trisinuate. *Eyes* large, with brownish-black colour, not distinct, 0.1 times as wide as cephalon. *Coxae* 1–7 visible in dorsal view, extending beyond their corresponding pereonites, gradually increasing the length and width, produced posteriorly, apex acute. *Pereonite* 1 shortest, anterolateral corners slightly produced; pereonites 1–5 gradually increase in width; pereonites 5 widest, 5 and 6 subequal; pereonite 7 shorter than 6, postero-lateral corners strongly produced, acute. *Pleon* narrower than pereon, all pleonites visible in dorsal view, lateral margins darkly pigmented, not overlapped by pereonite 7; pleonites 1 and 2 with lateral processes; pleonite 1 longest and widest, lateral margin strongly produced posteriorly; pleonites 2–5 sub equal, lateral margins posteriorly directed. *Pleotelson* triangular, converging smoothly to a caudomedial point, 1.2 times wider than long.

*Antennula* distinctly stouter than the antenna, 8-articled, articles 4–8 setose, articles 2 and 3 long, articles 2–8 decreasing in width, extending beyond the posterior of eye. *Antenna* slender, decreasing gradually in width, 11-articled, article 5 longest, 5–11 with distal setae, antenna extending to the posterior of the pereonite 1. *Mandible palp* slender, proximal segment longest and widest, lateral margin with few robust setae (RS); mandibular incisor blunt, well developed. *Maxillula* with 2 small and 2 large recurved apical RS. *Maxilla* medial lobe with 1, lateral lobe with 2 hooked robust setae in ovigerous female. In non-ovigerous females, 2 hooked robust setae on both medial and lateral lobes. *Maxilliped* oostegial lobe with many plumose setae; palp with 2 apical and one lateral small recurved robust seta on article 3. In non-ovigerous females, maxilliped palp with 2 apical and 2 lateral recurved robust setae on article 3.

*Pereopods* 1–7 with weak lobes on antereo-proximal margin of dactylus, gradually increase in length, ischium short and stout; pereopod 1 short; pereopods 1–6 without marginal robust setae, dactylus falcate; pereopod 7 long, dactylus extend to posterior of the carpus, carpus and merus stouter than the other pereopods, 4 robust setae on the margin of propodus, 2 robust setae on the carpus and merus, a single robust seta on distal inferior margins of the ischium.

Pleopods not distinctly visible in dorsal view; pleopods 1–2, endopod without lobes, pleopods 3–5 with proximo-medial lobe; Pleopods 3 and 4 endopod with a small single fold; pleopod 5 endopod with large multiple folds. Uropod slender; exopod straight, tapering, darkly pigmented, elongate about 8 times longer than wide, 1.7–2.0 times longer than endopod; endopod apex narrowly rounded or obliquely truncate.

***Description of partially moulted male*** (Figs. 6, 7, 8, 9): *Body* 2.2 times as long as wide, widest at pereonite 6. *Cephalon* broadly rounded anteriorly, 1.3 times as wide as long, posterior margin trisinuate. *Eyes* large, distinctly visible, 0.2 times as wide as cephalon. *Coxae* 2–4 not visible in dorsal view; coxae 5–7 visible dorsally, long, produced posteriorly slightly beyond their pereonites, apex acute. *Pereonites* 1–4 narrower and slightly increase in width from 1–4 pereonites, pereonite 1 shortest; pereonites 5–7 abruptly broader, pereonite 6 widest and 2.5 times wider than pereonite 1, pereonite 5 slightly smaller than pereonite 6, pereonite 7 slightly smaller than pereonite 5. *Pleon* 1.2–1.5 times broader than first 4 pereonites and 1.0–1.3 times narrower than posterior 3 pereonites. All pleonites visible in dorsal view, not immersed in pereonite 7; pleonites 1 widest; pleonites 2–5 subequal; the ventral process of pleonites 1 and 2 broad. *Pleotelson* as long as wide, triangular, converging smoothly to a caudomedial point.

*Antennula* distinctly stouter than the antenna, 8–articled; a cluster of setae on 4–8 articles; article 3 longest; article decreasing in width. *Antenna* slender, decreasing gradually in width; 11–articled, 5th article longest, 3, 4, 7 and 11 articles with distal setae. *Mandibular* palp article 3 lateral margin with about 11–14 stout setae, article 2 with 2 setae. Maxillula with 3 apical robust setae. *Maxilla,* medial lobe with 2 robust setae, lateral lobe with 1 robust seta. *Maxilliped* article 3, with 2 apical and 1 lateral small robust seta.

*Pereopods* 1–7 prehensile with weak swelling on antereo-proximal margin of dactylus, gradually increase in length, ischium short and stout, carpus immersed in merus; pereopod 1 short, dactylus reaching up to posterior of the merus; pereopods 1–6 without marginal robust setae, dactylus falcate; pereopod 7 long, dactylus extend to posterior of the carpus, carpus and merus stouter than the other pereopods, 4 robust setae on the margin of propodus, 2 robust setae on the carpus and merus, a single robust seta on distal inferior margins of the ischium.

*Pleopods* not distinctly visible in dorsal view; pleopods 1 and 2, endopod without lobes, pleopods 3–5 with proximo–medial lobe; pleopod 2 with appendix masculina about half the length of endopod; pleopods 3 and 4 endopod with a single small fold; pleopod 5 endopod with large multiple folds. *Uropodal* rami slender, long; exopod 1.3–1.4 times longer than endopod, overreaching telson.

**Colour.** Pale tan with chromatophores along posterior of pereon and pleon segments and lateral margin of uropod peduncle.

**Size.** Ovig. females: 17–26 mm, non ovig. females: 19–25 mm, moulting stage: 15–18 mm.

**Host and distribution:** list of all the recorded host fishes of *Nerocila phaiopleura* and its global distribution is provided in Table 1. The present materials are collected from *Dussumieria acuta* Valenciennes, 1847 and *Rastrelliger kanagurta* Cuvier, 1816, off south-west coast of India.

**Table 1:** List of all the recorded host fishes of *Nerocila phaiopleura* and its global distribution

Host fish		Distribution	References
Host family	Host species		
Chirocentridae	*Chirocentrus dorab* (Forsskål, 1775)	South Africa Kuwait India	Barnard (1925) [24] Bowan and Tareen (1983) [25] Ravichandran et al. (2001) [26] Trilles et al. (2011 and 2013) [2, 13]
	*Chirocentrus nudus* (Swainson, 1839)	Pakistan India	Ghani (2003) [27] Raja et al. (2014) [23]
	*Chirocentrus* sp.	Thailand	Bruce and Harrison-Nelson (1988) [28]
Clupeidae	*Clupea* sp.	Thailand	Bruce and Harrison-Nelson (1988) [28]
	*Konosirus punctatus* (Temminck & Schlegel, 1846)	Japan	Mitani (1982) [29]
	*Sardinella albella* (Valenciennes, 1847)	Indonesia China India	Bleeker (1857) [30] Morton (1974, as *S. perforata*) [31] Bharadhirajin et al. (2014) [32] Trilles et al. 2011 [13]
	*Sardinella brachysoma* (Bleeker, 1852)	India	Trilles et al. 2011 [13]
	*Sardinella fimbriata* (Valenciennes, 1847)	Thailand	Bruce and Harrison-Nelson (1988) [28]
	*Sardinella gibbosa* (Bleeker, 1849)	Indonesia China India	Bleeker (1857) [30] Morton (1974) [31] Trilles et al. 2011 [13] Trilles et al. (2013) [2]
	*Sardinella longiceps* (Valenciennes, 1847)	India	Trilles et al. (2011) [13] Trilles et al. (2013) [2] Rameshkumar et al. (2016) [33]
	*Sardinella sindensis* (Day, 1878)	India	Trilles et al. (2011) [13]
	*Sardinella zunasi* (Bleeker, 1854)	Japan China	Mitani (1982) [29] Bruce (1982, as *Harengula zunasi*) [34]
	*Sardinella* sp.	Thailand	Bruce and Harrison-Nelson (1988) [28]
	*Sardinops sagax* (Jenyns, 1842)	Japan	Mitani (1982) [29] Williams and Bunkley-Williams (1986) [35] Bruce and Harrison-Nelson (1988) [28] Hiramoto (1996) [36] Saito and Hayase (2000) [37] Nunomura (2011) [38] Hata et al. (2017) [39] Nagasawa et al. (2020, as *Sardinops melanostictus*) [40]
	*Tenualosa ilisha* (Hamilton, 1822)	India	Trilles et al. (2013) [2]
Dussumieriidae	*Dussumieria acuta* (Valenciennes, 1847)	Thailand Kuwait India	Monod (1934) [41] Bowman and Tareen (1983) [25] Trilles et al. (2011) [13] Trilles et al. (2013) [2] Ravichandran and Rameshkumar (2014) [42]
	*Dussumieria elopsoides* (Bleeker, 1849)	China	Morton (1974, as *D. hasselti*) [31]
	*Etrumeus micropus* (Temminck & Schlegel, 1846)	Japan	Nagasawa and Isozaki (2017) [12]
Engraulidae	*Engraulis australis* (White, 1790)	Australia	Bruce (1987) [3]
	*Engraulis japonicus* (Temminck & Schlegel, 1846)	Japan	Mitani (1982) [29] Bruce and Harrison-Nelson (1988) [28]
	*Engraulis sp.*	Thailand	Bruce and Harrison-Nelson (1988) [28]
	*Stolephorus commersonnii* (Lacepède, 1803)	India	Rajkumar et al. (2006, 2007, as *S* *commersonii*) [43, 44] Trilles et al. (2011) [13]
	*Stoleophorus indicus* (van Hasselt, 1823)	Indonesia	Bruce (1987) [3]
	*Thryssa dussumieri* (Valenciennes, 1848)	India	Trilles et al. (2011) [13] Bharadhirajin et al. (2014) [32]
	*Thryssa malabarica* (Bloch, 1795)	India	Aneesh et al. (2013) [10]
	*Thryssa mystax* (Bloch & Schneider, 1801)	India	Trilles et al. (2011) [13] Aneesh et al. (2013) [10] Trilles et al. (2013) [2]
	*Thryssa setirostris* (Broussonet, 1782)	India	Aneesh et al. (2013) [10]
Pristigasteridae	*Illisha melastoma* (Bloch & Schneider, 1801)	Singapore India	Bruce and Harrison-Nelson (1988) [28] Trilles et al. (2011) [13]
	*Ilisha filigera* (Valenciennes, 1847)	India	Trilles et al. (2011) [13]
	*Opisthopterus tardoore* (Cuvier, 1829)	India	Trilles et al. (2011) [13] Aneesh et al. (2013) [10]
*Mugilidae*	*Chelon parsia* (Hamilton, 1822)	India	Bharadhirajin et al. (2014, as *Liza parsia*) [32]
	*Gracilimugil argenteus* (Quoy & Gaimard, 1825)	Indonesia, Australia	Bruce (1987, as *Liza argentea*) [3]
Ariidae	*Arius jella* (Day, 1877)	India	Trilles et al. (2011) [13]
Plotosidae	*Cnidoglanis macrocephalus* (Valenciennes, 1840)	Australia	Bruce (1987, as *Cnidoglannus macrocephalus*) [3]
*Carangidae*	*Carangoides malabaricus* (Bloch & Schneider, 1801)	India	Trilles et al. (2013) [2]
	*Carangoides sp.*	India	Trilles et al. (2013) [2] Rameshkumar et al. (2016) [33]
	*Decapterus maruadsi* (Temminck & Schlegel, 1843)	China	Morton (1974) [31]
	*Parastromateus niger* (Bloch, 1795)	India	Bruce and Harrison-Nelson (1988) [28] Trilles et al. (2011) [13]
	*Selaroides leptolepis* (Cuvier, 1833)	India	Trilles et al. (2013) [2]
	*Trachurus japonicus* (Temminck & Schlegel, 1844)	Japan	Nagasawa and Isozaki (2017) [12]
Istiophoridae	*Istiophorus platypterus* (Shaw, 1792)	India	Barnard (1936, as *Histiophorus gladius*) [45] Trilles et al. (2011) [13]
Leiognathidae	*Eubleekeria splendens* (Cuvier, 1829)	India	Trilles et al. (2013, as *Leiognathus splendens*) [2]
	*Gazza minuta* (Bloch, 1795)	India	Trilles et al. (2013) [2]
Polynemidae	*Polynemus* sp.	Indonesia	Trilles (1979) [46]
Scombropidae	*Scombrops boops* (Houttuyn, 1782)	Japan	Nagasawa and Tensha (2016) [47] Nagasawa and Isozaki (2020) [48]
	*Scombrops gilberti* (Jordan & Snyder, 1901)	Hibiki-Nada Sea, Japan	Masakazu et al. (2021) [49]
Scombridae	*Rastrelliger kanagurta* (Cuvier, 1816)	India	Rameshkumar and Ravichandran (2010) [50] Trilles et al. (2011 and 2013) [13] [2] Seth et al. (2014) [51] Amrutha et al. (2021) [22] Ramudu and Rathod (2023, 2024) [52, 53] Present study
	*Scomber japonicus* (Houttuyn, 1782)	Japan	Nagasawa and Nakao (2017) [54]
	*Scomberomorus guttatus* (Bloch & Schneider, 1801)	India	Trilles et al. (2011) [13]
	*Scomberomorus niphonius* (Cuvier, 1832)	Japan	Nagasawa and Tensha (2016) [47] Hata et al. (2017) [39]
	*Thunnus orientalis* (Temminck & Schlegel, 1844)	Japan	Nagasawa and Shirakashi (2017*)* [55]
Sphyraenidae	*Sphyraena japonica* (Bloch & Schneider, 1801)	Japan	Nagasawa and Isozaki (2017) [12]
	*Sphyraena jello* (Cuvier, 1829)	India	Trilles et al. (2013) [2]
Priacanthidae	*Priacanthus hamrur* (Forsskål, 1775)	India	Jalaja Kumari et al. (1987) [56]
Dorosomatidae	*Amblygaster sirm* (Walbaum, 1792)	India	Trilles et al. (2011) [13]
	*Nematalosa nasus* (Bloch, 1795)	India	Trilles et al. (2011) [13]
Nemipteridae	*Nemipterus japonicus* (Bloch, 1791)	India	Trilles et al. (2011) [13]

**Ecological remarks:** The cymothoid, *N. phaiopleura,* was found attached firmly to the posterior third of the body, overlying the lateral line and facing the head of the host fish (Fig. 10a, b). Discrete alterations such as haemorrhages, loss of scales, and extensive skin erosions/skin ulceration were gross pathological symptoms of the attachment site of the parasite. On the body surface, small pinholes with the formation of epidermal plaques (Fig. 10c, d) and large, round-shaped haemorrhagic wounds/ulcers (no fish skin was present on the wounds where the muscle was exposed) (Fig. 10e) were noticed.

**Molecular analysis:** 100% similar COI sequences for *N. phaiopleura* were generated when submitted to GenBank with GenBank accession number OP890359. The COI sequence was compared to other known *Nerocila* spp. sequences available on GenBank (Table 2). The alignment was 647 bp, codon positions included were 1st + 2nd + 3rd, all positions containing gaps and missing data were eliminated. Nucleotide genetic divergence (p-distance) among *N. phaiopleura* and other available *Nerocila* spp. ranged from 21 to 29%. The P genetic distance was high (29%) between *N. phaiopleura* and *N. longispina.* The genetic distance was less (21%) between *N. phaiopleura* and *N. poruvae*.

**Table 2:** P-distance of sequences from *Nerocila phaiopleura* and *Nerocila* spp. available in GenBank based on COI gene sequences

	1	2	3	4	5	6	7
1. OP890359 *Nerocila phaiopleura*							
2. KY933655 *Nerocila loveni*	28						
3. OK001962 *Nerocila longispina*	35	22					
4. LC160331 *Nerocila japonica*	26	23	16				
5. ON661340 *Nerocila exocoeti*	28	2	19	18			
6. MZ644982 *Nerocila orbignyi*	29	28	28	23	23		
7. EF455819 *Nerocila bivittata*	30	26	26	32	24	27	

The code indicated for each species refer to the accessed code in Genbank

## Discussion

*Nerocila phaiopleura* Bleeker, 1857, is redescribed here in detail based on the specimens collected from the Southwest coast of India. The results revealed that the cymothoid, *N. phaiopleura,* could be identified by the following combinations of characters: body about 2.4 times as long as wide; cephalon broadly rounded anteriorly; coxae posteriorly directed, acute and extending beyond their corresponding pereonites; pereonites 7 posterior angle produced, extending to the pleonite 1; pleonites 1 and 2 ventrolateral process posteriorly directed; uropod exopod straight and elongate about 8–9 times longer than proximal width, 1.7–2.0 times longer than endopod; uropod endopod lateral margin not serrate; no notch on medial margin; pleotelson triangular and the distinctive pereopod morphology (e.g. pereopods with a short ischium). *Nerocila phaiopleura* differs from the closely related species *N. depressa* by the coxae, and posterolateral comers of the pleonites are posteriorly directed and are not bent dorsally in *N. phaiopleura* (vs the coxae and posterolateral comers of the pleonites are slightly bent and directed dorsally in *N. depressa*); the uropod endopod with an obliquely truncate apex in *N. phaiopleura* (refer Figs. 1, 2, 3, 4, 5).

The marked difference between the male moulting stage and the female is that the body shape of the male moulting stage is not oval; instead, the body is abruptly narrower anteriorly (anterior-most pereonites 1–4 are abruptly narrower than the remaining pereonites). The variations shown by the male moulting stage from the female are: pereonite 5, 2 times wider than pereonite 4 (in females, pereonite 5, 1.1 times wider than pereonite 4); pleon about 1.4 times broader than first 4 pereonites and about 1.2 times narrower than posterior 3 pereonites (in females, pleon narrower than pereon); mandibular palp article 3 lateral margin with more (about 11–14) stout setae; robust setae on pereopods 1–4 and 7 (in females, robust setae present on pereopod 7 only) (refer Figs. 1, 2, 3, 4, 5, 6, 7, 8, 9).

The previous description of *N. phaiopleura* are short and do not consider most of the taxonomic details. The male-moulting stage of *N. phaiopleura* is not characterized yet. The general morphology and appendages of life cycle stages (transitional (partially moulted) and ovigerous female) of Indian specimens are described and illustrated here. Key taxonomic features provided for the proper identification of *N. phaiopleura*.

Variations in the morphology of the body parts were observed in the present Indian specimens of *N. phaiopleura* compared to Australian samples described by Bruce [3]. The mandible palp article lateral margin has about 22 stout setae in the descriptions provided by Bruce [3], whereas the present specimens have mandible palp article lateral margin without setae or with a few setae. Pereopod 7 has 2 spines on the posterior margin of the propodus, according to Bruce [3], whereas in the present study, pereopod 7 has 4 spines on the margin of the propodus, 2 spines on the carpus and a single spine on the distal inferior margins of the ischium. Illustrations and descriptions of the other morphological characters such as maxilla, maxillula, 1st and 2nd antenna, pereopod 2–6, and pleopods of ovigerous females were not included in the descriptions provided by Bruce [3].

The comparison of genetic distances among *N. phaiopleura* and the available *Nerocila* spp. on GenBank was provided based on COI sequences (refer to Table 2). The genes (COI sequences) of *Nerocila* spp. can provide more information on genetic distances among species, which can be linked with their morphological aspects. Integrative taxonomy for *Nerocila* can aid a better understanding of the species, as in the case of the present study, drawing more conclusions on the actual diversity and distribution of the genus.

The isopod parasite, *N. phaiopleura,* causes discrete alterations such as haemorrhages, loss of scales, and extensive skin erosions/ ulceration at the attachment site. On the body surface, small pinholes with the formation of epidermal plaques and large, round-shaped haemorrhagic wounds/ulcers were observed as gross pathological symptoms (refer Fig. 10). Amrutha et al. [22] observed that, at the insertion site of the parasite’s pereopods, there was deep skin depression along with massive lymphocyte infiltrations. The epidermis around the pereopod attachment site was hyperplasic, clearly manifesting epidermal spongiosis. The host response includes infiltration of inflammatory cells, primarily macrophages, lymphocytes and eosinophils. Muscle fibrosis and degeneration, along with oedema and tissues undergoing necrosis, were evident in the muscle tissues. The significant histopathological changes in the fish tissues caused by *N. phaiopleura* are probably due to attachment, movement and feeding. Furthermore, localized loss of osmoregulatory skin function may occur at the lesions. Finally, the isopods may mechanically impede swimming performance, making infested fish more susceptible to predation and indirectly causing increased mortality in wild fish populations.

*Nerocila phaiopleura* has a wide geographical distribution and host range. It is reported from 50 species of fish belonging to 14 families, including Clupidae, Engraulidae, Carangidae, Scombridae, Dussumieriidae, Chirocentridae, Pristigasteridae, Mugilidae, Sphyraenidae, Leiognathidae, Plotosidae, Polynemidae, Ariidae and Istiophoridae from the Indian Ocean and Pacific Ocean [8, 9, 12, 23]. This clearly shows that this species is the least host-specific and can potentially threaten the wild and farming of fish. Almost all host fish are important and form a significant catch in commercial ladings in many countries, including India.

## Data Availability

No datasets were generated or analysed during the current study.

## Abbreviations

RS: Robust seta/e
TL: Total length
W: Width
